# 1444. Candida auris Outbreak in a COVID-19 Hospital: Detection and Containment

**DOI:** 10.1093/ofid/ofad500.1281

**Published:** 2023-11-27

**Authors:** Victor Baylon-Valdez, Gloria Mayela Aguirre-García, Claudia E Guajardo-Lara, Mary C Aleman-Bocanegra, Michel F Martínez-Reséndez

**Affiliations:** Instituto Tecnológico y de Estudios Superiores de Monterrey, School of Medicine and Health Sciences, Monterrey, Nuevo Leon, Mexico, Monterrey, Nuevo Leon, Mexico; Instituto Tecnológico y de Estudios Superiores de Monterrey, School of Medicine and Health Sciences, Moonterrey, Nuevo Leon, Mexico; Instituto Tecnológico y de Estudios Superiores de Monterrey, School of Medicine and Health Sciences, Monterrey, Nuevo Leon, Mexico, Monterrey, Nuevo Leon, Mexico; Instituto Tecnológico y de Estudios Superiores de Monterrey, School of Medicine and Health Sciences, Monterrey, Nuevo Leon, Mexico, Monterrey, Nuevo Leon, Mexico; Instituto Tecnológico y de Estudios Superiores de Monterrey, School of Medicine and Health Sciences, Monterrey, Nuevo Leon, Mexico, Monterrey, Nuevo Leon, Mexico

## Abstract

**Background:**

*Candida auris* has become an important nosocomial infection in ICU patients. The difficulty in isolating and detecting this pathogen, its inherent multidrug resistance, and rapid transmission make it a menace to public health. The present work details the first *C. auris* outbreak in Mexico and containment techniques used in a Tertiary Care Center that underwent restructuring due to COVID-19.

**Methods:**

We performed a single-center, retrospective, descriptive study from June 2020 to February 2022 at Hospital San Jose TecSalud, Monterrey, including hospitalized COVID-19 patients with detection of *Candida auris* by MALDI-TOF. After identifying the first cases, the exposed patients were screened twice weekly by skin (armpit/groin) swab culture. Those who tested positive were classified as colonization. Patients with clinical signs of infection were tested from sterile sites (blood/urine), and if the culture came back positive they were classified as cases. Finally, we described the interventions for containment.

**Results:**

During the study period, 4,025 patients were hospitalized due to COVID-19; 971 were critically ill patients admitted to the ICU. 160 patients tested positive for *Candida auris*, 131 colonizations, and 29 cases (Figure 1). Of the total, 83% were male, mean age ± SD = 56.96 ± 13.29. The most common sites of infection in the cases group were urinary tract infections (55.17%) and bloodstream infections (44.81%; from this, 23% were catheter-associated). The first case was detected on June 4th, 2020, in July 2020, we transitioned to a single patient-per-nurse model and implemented disinfection validation via ATP bioluminescence. Also, to control the increase in cases, by the end of 2020, we standardized chlorine cleaning and de-escalated PPE, and implemented hydrogen peroxide vapor and reinforcement of hand-washing techniques (Figure 2).

**Figure 1.**
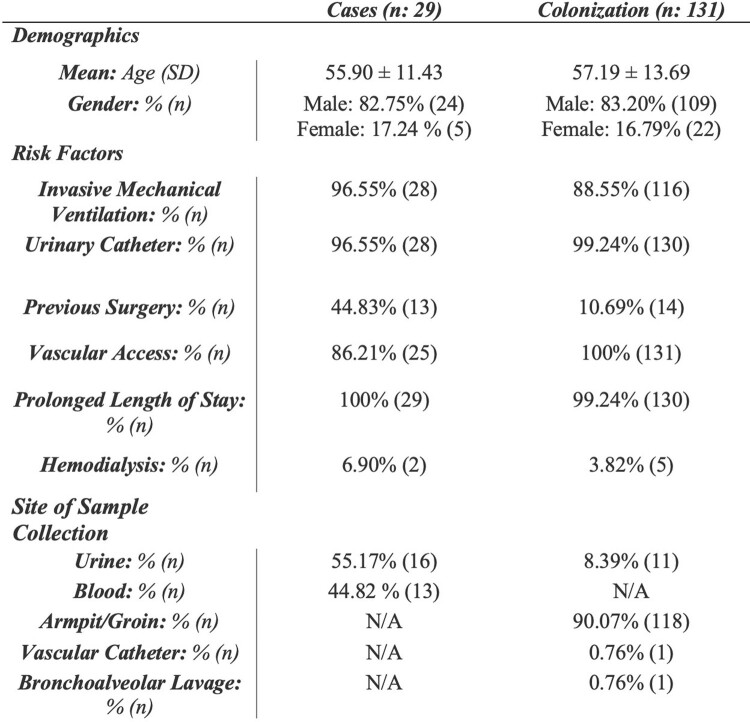
Patient’s Demographics.

**Figure 2.**
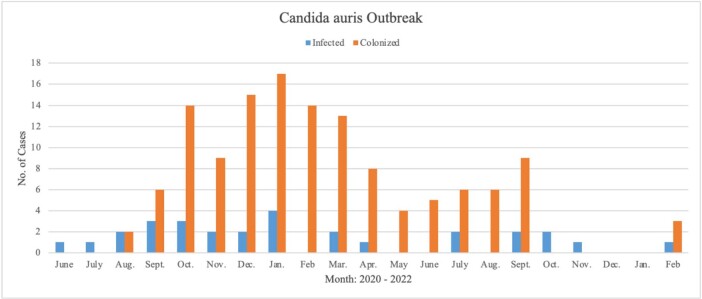
Outbreak.

**Conclusion:**

In the setting of the transition of Hospital San Jose into a COVID-19 Hospital in response to the high volume of patients, and the emergence of *C. auris* at the ICU; early detection and screening of the exposed population, multi-disciplinary coordination between healthcare teams, and the rapid installation of interventions were crucial for the containment and termination of the outbreak.

**Disclosures:**

**All Authors**: No reported disclosures

